# Geospatial Imprecision With Constraints for Precision Public Health: Algorithm Development and Validation

**DOI:** 10.2196/54958

**Published:** 2024-05-21

**Authors:** Daniel Harris, Chris Delcher

**Affiliations:** 1 Institute for Biomedical Informatics University of Kentucky Lexington, KY United States; 2 Department of Pharmacy Practice and Science College of Pharmacy University of Kentucky Lexington, KY United States

**Keywords:** social determinants of health, geocoding, privacy, poverty, obfuscation, security, confidentiality, low income, geography, geographic, location, locations, spatial, geospatial, precision

## Abstract

**Background:**

Location and environmental social determinants of health are increasingly important factors in both an individual’s health and the monitoring of community-level public health issues.

**Objective:**

We aimed to measure the extent to which location obfuscation techniques, designed to protect an individual’s privacy, can unintentionally shift geographical coordinates into neighborhoods with significantly different socioeconomic demographics, which limits the precision of findings for public health stakeholders.

**Methods:**

Point obfuscation techniques intentionally blur geographic coordinates to conceal the original location. The pinwheel obfuscation method is an existing technique in which a point is moved along a pinwheel-like path given a randomly chosen angle and a maximum radius; we evaluate the impact of this technique using 2 data sets by comparing the demographics of the original point and the resulting shifted point by cross-referencing data from the United States Census Bureau.

**Results:**

Using poverty measures showed that points from regions of low poverty may be shifted to regions of high poverty; similarly, points in regions with high poverty may be shifted into regions of low poverty. We varied the maximum allowable obfuscation radius; the mean difference in poverty rate before and after obfuscation ranged from 6.5% to 11.7%. Additionally, obfuscation inadvertently caused false hot spots for deaths by suicide in Cook County, Illinois.

**Conclusions:**

Privacy concerns require patient locations to be imprecise to protect against risk of identification; precision public health requires accuracy. We propose a modified obfuscation technique that is constrained to generate a new point within a specified census-designated region to preserve both privacy and analytical accuracy by avoiding demographic shifts.

## Introduction

Geographic information systems (GISs) are increasingly important for public health research and policy makers and are instrumental in measuring socioeconomic equity in health care [1,2]. Social determinants of health are the conditions in which individuals are born, live, work, and age; mesolevel determinants are from the physical environment and encompass items such as geographic location and access to resources [3]. Location-based exposures tied to geographic location are a pivotal element to one’s health [4-6]; ongoing research suggests that zip code is on par with genetic code in influencing individual health [7-10]. Even greater research utility lies in more precisely geolocating patients beyond the zip code level, yet privacy regulations often prevent high-resolution patient residential address data from being shared for research purposes [11]. Privacy is paramount when working with health care data and access is regulated at different levels through both institutional policies and government-mandated legal protections [12]. The Health Insurance Portability and Accountability Act (HIPAA) mandates privacy protections of personal health information in the United States; it outlines which data elements are considered private, including patient addresses needed for geospatial analysis for place-based epidemiology.

Although institutional review boards may grant researchers and other agencies access to identified data that pose minimal risk to the patient, there may exist institutional hesitancy to disclose this data due to the inherent privacy and sensitivity of residential addresses. As a current example, this tension is apparent between state and federal public health and safety agencies using the Overdose Detection Mapping Application Program (ODMAP), which maps in real time the exact locations of suspected drug overdoses, often occurring in residential locations [13,14]. Geocoding a patient’s address (ie, converting to geographic coordinates) is often an intermediate step in secondary data analyses; it is either used to link the patient to external geographic units (eg, census tracts to obtain neighborhood socioeconomic status from the United States Census Bureau) or to calculate distance from other entities, such as health care providers and facilities. For example, accessibility of buprenorphine, a medication for opioid use disorder, may be determined using addresses of health care providers that are authorized to prescribe the medication [15]. In these examples, imprecise locations may be sufficient for confident linkage to administrative units or approximate distance measures and preferred for research to preserve privacy. In these scenarios, thoughtful and controlled techniques designed to generate inexact data are needed to reduce precision [12,16].

To this end, geospatial or location-based privacy methods seek to maintain an appropriate level of confidentiality for a given task, service, or application while balancing the utility that these offer [17-19]. For example, users of location-based services on a cellular phone expect some level of privacy when sending personal data, and different strategies exist that anonymize pools of people by anonymizing data at point of collection. Location-based *k*-anonymity provides a method where one’s data and location are indistinguishable from *k*–1 other people [20]. Other methods, such as geographic masking, alter coordinates systematically to limit the risk of reidentification when releasing data [21]; no universally accepted method exists for protecting geospatial privacy [16].

**Figure 1:**
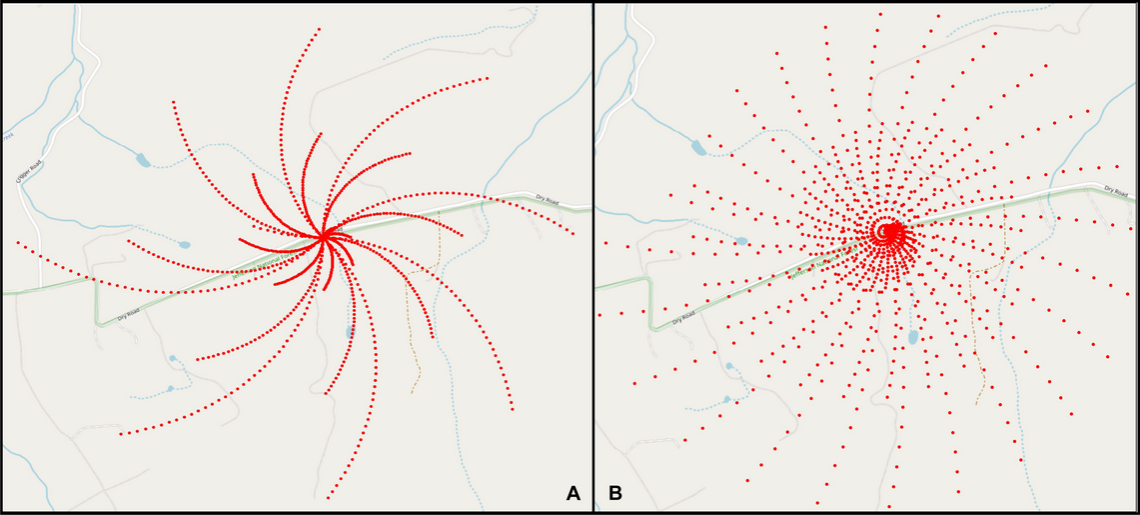
Pinwheel obfuscation at theta 45 degrees (A) and 15 degrees (B).

Point obfuscation refers to the deliberate degrading of the resolution of coordinate information with the goal of protecting the privacy of the individual represented by the point [22]; this may be referred to as geographic masking, geomasking, jittering, or dithering and relies upon transformations or perturbations using randomness or artificial noise [22]. The *N-RAND* algorithm generates *N* candidate points in a given area and selects the furthest point [23]; *theta-RAND* limits candidate points to a specific area defined by a chosen angle [24]. We introduce modifications to the pinwheel obfuscation method which shifts points along pinwheel-like paths for a randomly chosen angle [25]; examples are shown in Figure 1. The noise added by this method is asymmetrical and highly variable, making it less open to privacy attacks designed to eliminate uniform and predictable noise [25]. However, the limitation of any point obfuscation technique is that coordinate shifts may change real-world locations and distort the linked health-related metrics. For example, a study defining a participant’s rurality based on administrative units may be impacted if obfuscated coordinates move the participant across boundaries into an urban area.

Address *correction* positively impacts the accuracy of assigning a patient to a geographic area [26]. On the other hand, the goal of point obfuscation is to *intentionally generate an incorrect* address to preserve patient privacy without compromising analytical conclusions. Our paper demonstrates that indiscriminate point obfuscation impacts studies linking points to neighborhood-level socioeconomic demographics and subsequently provides new methods needed to constrain pinwheel obfuscation to yield results confined in specific census-designated regions, such as blocks, block groups, or tracts. The constraint reduces concerns that neighborhood-level measures are inappropriately assigned at the patient level, leading to misclassification bias. We use poverty status as an example of data recorded by the United States Census Bureau to explore the potential impact of unintentional administrative boundary shift. Also, we demonstrate how indiscriminate point obfuscation impacts hot spot analysis at the census tract level. The role of this work is to provide evidence that point obfuscation techniques may substantially alter neighborhood-level socioeconomic demographics and that the intentional imprecision in these techniques must be constrained to support precision public health.

## Methods

### Overview

We implemented our methods using PostGIS, an open-source project that adds geospatial objects and procedures to the PostgreSQL database. We previously demonstrated PostGIS as a capable environment for geospatial privacy research [27,28]. We make our custom PostGIS functions available as open-source software [29]. The pinwheel technique was originally designed because other point obfuscation methods could be reversed by methods designed to filter uniform noise; the randomness of the pinwheel has been shown to maintain high variability, making it less susceptible to privacy attack [25]. Our geographically constrained pinwheel algorithm leverages the same concept as the original pinwheel algorithm and improves its research utility by adding constraint checking that controls how the new obfuscated point is selected. There is a function, *PINWHEEL*, that obfuscates a single point given a specific *theta*, maximum radius *r*, and a calculated random degree *a*; these are used to calculate a projection distance that can leverage PostGIS’s projection function, *ST_PROJECT*, to calculate the resulting obfuscated point.

The left-hand side of Figure 1 shows 1000 candidate points for a given seed point using a 45-degree angle; we sequentially varied the maximum radius while keeping *theta* constant at 45 degrees. Similarly, the right-hand side of Figure 1 shows the same simulation but with 15-degree angles; this demonstrates that *theta* controls the width of the pinwheel layers and that small angles naturally yield closer layers. In practice, a random degree may be used to further obfuscate the results.

Census Bureau geometries are input as reference data. The smallest census-based geographic boundary, the census block, is contained in the block group; a census block group typically represents between 600 and 3000 people. Block groups are organized into census tracts, which typically contain between 1200 and 8000 people and have an optimum size of 4000 people [30]. The United States Census Bureau publishes and updates files containing the geometries for these regions; these geometries are used in point intersection calculations to assign a region to a given point.

To obfuscate, points from the original list, *P1*, are fed into the *PINWHEEL* function and saved into *P2*. To constrain obfuscation, our method recalculates the pinwheel candidate for any generated candidates falling outside a specific region associated with the original point; we can constrain to standard administrative units: state, county, tract, block group, and block geometry. Furthermore, we can constrain obfuscation to custom geometries, such as buffer zones or other areas that may be relevant for research projects. The region of the points can be calculated with PostGIS’s *ST_CONTAINS* function, which tests the intersection of the points with the Census Bureau geometries. The regions are compared for every matching pair of existing and new points in *P1* and *P2*. If the regions are dissimilar, *PINWHEEL* is rerun on the existing point. This continues until all points in *P1* have a matching obfuscated point in *P2* where *P1* regions align with *P2* regions.

We tested our methods with 2 different data sets, with geographic coverage ranging from multiple states to a single large urban area. Our first data set contained 1,000,000 records formatted in the Observational Medical Outcomes Partnership (OMOP) common data model [31]; we previously leveraged this data for geospatial research on open data and privacy [27]. Our second data set contains 58,102 case records from the Medical Examiner Case Archive from Cook County, Illinois, which contains the city of Chicago; we previously used this open data for research on geographic clustering of fatal overdoses [32] and to create an open data pipeline for spatial analyses on substance use disorders [33]. This open data set was released by the Cook County Medical Examiner’s Office (CCMEO) and offers details on all deaths recorded by the CCMEO from August 2014 to April 2022, including the address where the incident occurred and the address of the death. Deaths by suicide recorded in the CCMEO data set were also used for our hot spot analysis example.

### Ethical Considerations

This study was exempt from ethical review since no private health data were used and no human subjects were involved.

## Results

As expected, the pinwheel obfuscation method initially resulted in points shifting into different geographic regions; the frequency of these region shifts is summarized in Table 1 for our OMOP and CCMEO data. We categorized these shifts by census-designated regions by increasing population size (block, block group, tract, county, and state). Shifts were proportional to the maximum radius allowed; a radius of 1000 meters was the largest distance tested and naturally generated the most region shifts. Blocks are the smallest geographic unit and experienced the most change: 747,934 of 1,000,000 (74.8%) of the OMOP points and 53,415 of 58,102 (91.9%) of the CCMEO points were moved to different census blocks after obfuscation up to 1000 meters away. This empirically indicates that smaller geographical regions are more likely to shift when using any significant distance in the obfuscation method. Blocks are the smallest of the census-designated administrative boundaries. There is no maximum size for a census block; minimum block size is 30,000 square feet (2787.1 m^2^) for polygons bounded by roads or 40,000 square feet (3716.1 m^2^) otherwise, which is smaller than the largest obfuscation distance selected for our testing [34].

Additionally, the results demonstrate that even with very small distances unintentional consequences may occur; even moving the point using a radius of 1 meter resulted in misclassification. Although relatively rare, point obfuscation 1 meter away could change a point’s county in 0.0007% of the OMOP data (7/1,000,000) or a point’s census tract in 0.09% of the CCMEO data (58/58,102). There are no universally accepted best practices for point obfuscation, and the most effective allowed distance may vary with study area and application [16]. Due to the inclusion of the city of Chicago in the Cook County data, the census-designated regions in the CCMEO data are geographically smaller than those in our OMOP data, which cover multiple states; census tracts generally contain between 1200 and 8000 residents, meaning urban census tracts are geographically smaller than rural census tracts. This can be seen in our results, where a radius of 100 meters or larger yielded a higher percentage of shifts in our CCMEO data than our OMOP data.

Our results demonstrate that indiscriminate point obfuscation can shift a point into different census-designated geographical regions; this is a natural and expected consequence of moving a point. However, we now discuss and quantify the potential impact of shifting to linked administrative units (ie, neighborhoods) by comparing census demographics before and after obfuscation. The United States Census Bureau conducts large-scale surveys, such as the decennial census and the American Community Survey (ACS). The yearly ACS samples approximately 250,000 household units monthly. From the ACS, we picked the estimated number of “individuals with income in the past 12 months below poverty level” as an example demographic; these data are publicly available at the census tract level. We chose poverty status due to its saliency in health outcomes research [35,36].

We give a high-level overview of the obfuscation impact on poverty status measurement in Table 2 to justify the need for a geographically constrained obfuscation technique when assigning points to a population-based rate of individuals living under the poverty line (as a percentage of the total population). In our OMOP data, a pinwheel distance of 1000 meters resulted in 22.4% (n=224,065) of records with a different poverty rate after obfuscation where those changes were, on average, a mean 7.3% (SD 7.4%) away from the original rate (median 5%, range 91.9% to –78.6%). We also show the magnitude of the difference between the original and obfuscated address by showing minimum and maximum differences of rates. Negative differences imply the obfuscated record had a lower assigned poverty rate while positive differences imply the obfuscated record had a higher assigned poverty rate. For completeness and to complement Table 1, we include small distances of 10 and 1 meters in Table 2, although we did not anticipate such small distances would impact poverty rate assignments.

**Table 1:** The number of records shifted into a different region varied per obfuscation radius (in meters) and size of region.

Data set and radius (meters)			Records, n×1000 (%)								
			Block		Block group		Tract		County		State
**Observational Medical Outcomes Partnership (n=1,000,000)**											
	1000	747 (75)		367 (37)		226 (22)		16 (1.6)		1.1 (0.11)	
	500	616 (62)		210 (21)		114 (12)		8 (0.8)		0.479 (0.049)	
	100	236 (24)		35 (3.5)		16 (1.6)		1.3 (0.13)		0.071 (0.007)	
	1	3.2 (0.34)		0.359 (0.03)		0.194 (0.02)		0.08 (0.008)		0.004 (0.0004)	
	1	0.304 (0.03)		0.044 (0.004)		0.018 (0.001)		0.007 (0.0007)		0 (0)	
**Cook County Medical Examiner’s Office (n=58,102)**											
	1000	53 (91.9)		40 (70.4)		31 (53.3)		0.161 (0.27)		0.034 (0.05)	
	500	49 (85.4)		29 (50.7)		19 (33)		0.082 (0.14)		0.01 (0.02)	
	100	27 (47.7)		7.8 (13.4)		4.5 (7.8)		0.012 (0.02)		0.001 (0.002)	
	10	1.2 (2.22)		0.495 (0.85)		0.343 (0.59)		0.001 (0.002)		0 (0)	
	1	0.187 (.032)		0.082 (0.14)		0.058 (0.09)		0 (0)		0 (0)	

**Table 2:** Poverty rates are substantially different before and after obfuscation.

Data set and distance (meters)			Records with changed poverty rate, n (%)		Difference in rate (%), mean (SD)		Difference in rate (%), median (maximum to minimum)
**Observational Medical Outcomes Partnership (n=1,000,000)**							
	1000	224,065 (22.4)		7.3 (7.4)		5 (91.9 to –78.6)	
	500	113,682 (11.3)		7.3 (7.5)		5 (91.9 to –78.6)	
	100	16,121 (1.6)		7.4 (7.7)		5.1 (78.6 to –78.6)	
	10	193 (0.01)		6.5 (6.4)		4.2 (35.9 to –32.6)	
	1	18 (0)		11.7 (8.1)		10.5 (38.8 to –12)	
**Cook County Medical Examiner’s Office (n=58,102)**							
	1000	30,843 (53.1)		8.3 (7.7)		6 (61.7 to –69)	
	500	19,139 (32.9		8.2 (7.6)		5.9 (58.4 to –61.7)	
	100	4506 (7.8)		8.2 (7.5)		6 (58.4 to –58.4)	
	10	343 (0.6)		8.1 (7.3)		6.2 (33.2 to –55.4)	
	1	58 (0.1)		6.8 (6.5)		4.4 (31 to –18.2)	

A larger percentage of records in the CCMEO data experienced rate changes in comparison to our OMOP data. With a distance of 1000 meters, 53.1% (n=30,843) of records were assigned into a region with a different poverty rate where those changes were a mean 8.3% (SD 7.7%) away from the original rate (median 6%, range 61.7% to –69%). The magnitude of changes was not substantially different from our OMOP data (averages of 7.3% vs 8.3% and medians of 5% vs 6%, respectively, for 1000 meters); yet the frequency of these changes was notably higher (22.4% vs 53.1%, respectively, for 1000 meters).

**Figure 2:**
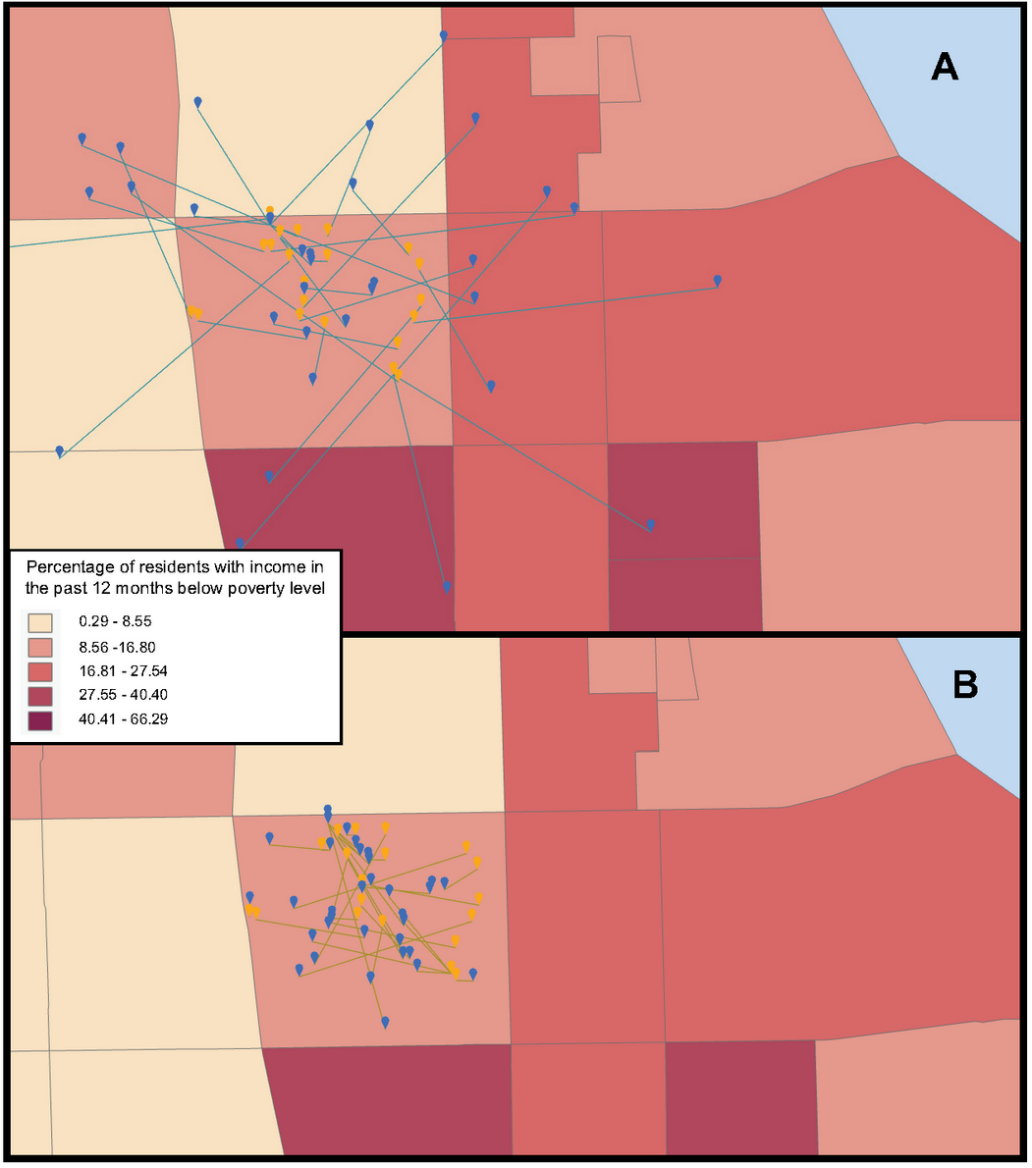
Poverty and point obfuscation of original points (orange) to obfuscated points (blue) using a pinwheel (A) and a geographically constrained pinwheel (B).

Figure 2 shows an example census tract (17031031100) in Cook County, Illinois; 33 deaths were recorded in this area. Figure 2A shows an example simulation using pinwheel obfuscation with a 1000-meter radius; Figure 2B shows the results of our geographically constrained pinwheel obfuscation. The original point is orange, and the obfuscated point is blue; the census tracts are colored according to quintile of our poverty measure, where lightly colored areas have the lowest poverty rates. For this example, pinwheel obfuscation resulted in 22 of 33 (66%) of the points shifting census tracts; 12 of 33 (36%) of these were shifted into areas of higher poverty, while 10 of 33 (33%) were shifted into areas of lower poverty. Of those positive shifts, 4 of 12 were pushed to the highest category (33%) while the other 8 were moved into the second-highest poverty quintile. This example shows how obfuscation may move points from one extreme to another.

**Figure 3:**
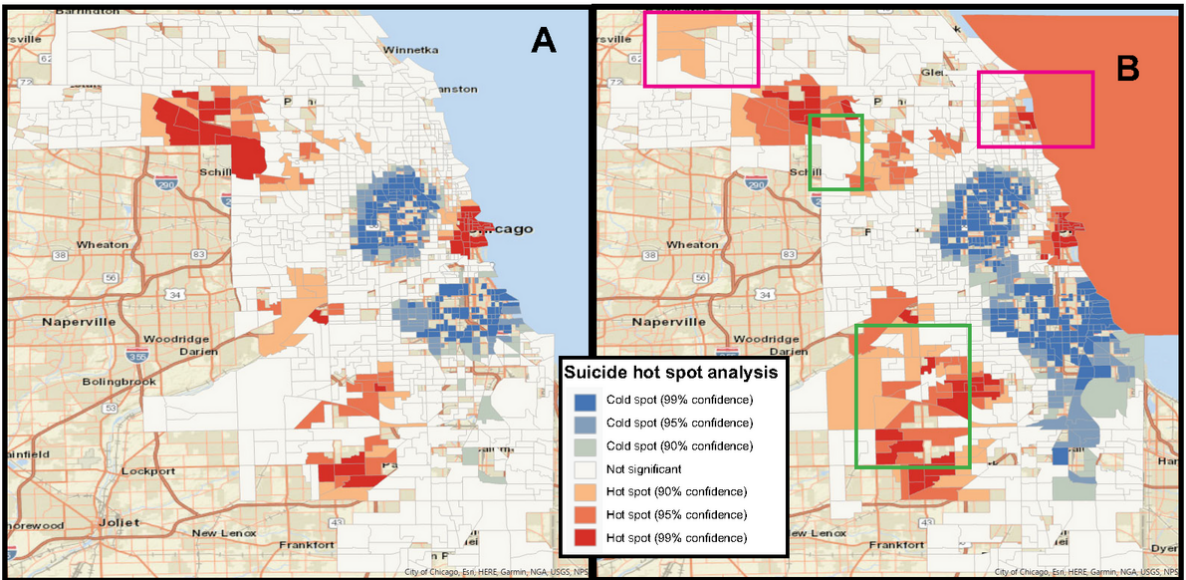
Hot spot analysis of suicides using original data (A) and obfuscated data (B).

Hot spot analysis is known to be an effective tool for understanding how health outcomes and social determinants of health concentrate and cluster together [37-39]. We explored the impact of indiscriminate point obfuscation on a hot spot analysis of deaths by suicide from our CCMEO data; suicides were identified by the manner of death field in the CCMEO data and span the time period August 2014 to April 2022. Figure 3 shows the results of hot spot analyses using ArcGIS Pro [40] on the original data (Figure 3A) and data obfuscated with the pinwheel method using an unconstrained 1000-meter radius (Figure 3B). The obfuscation naturally blurred the correct hot spots, but (unexpectedly) new hot spots emerged, as identified by the pink boxes in Figure 3B. Most notably, a hot spot (95% confidence) spills into the neighboring and uninhabited Lake Michigan (census tract 17031990000). Highlighted in green are regions of interest with substantial change; the upper green box demonstrates the disappearance of a hot spot (99% confidence), and the lower green box demonstrates how indiscriminate obfuscation can bridge 2 hot spots together and weaken the signal that distinct clusters exist. By definition, the hot spots corresponding to the geographically constrained pinwheel method are identical to the true clusters in Figure 3A because of the confinement to the point’s original census tract. When linked to an administrative boundary such as census tract, results are consistent before and after obfuscation when the pinwheel method is constrained; only distance-based results would be impacted by moving the original point. By constraining the pinwheel process to a specific geographic region, the results of any method depending upon aggregation within those regions will not be impacted by our method.

## Discussion

### Principal Findings

We demonstrated that imprecise point obfuscation results in shifts across geographic regions and showed that these shifts do result in points geolocating in regions with vastly different socioeconomic contexts. This justifies the need for more precise point obfuscation techniques; our method constrains the candidate points into a specific region, which guarantees identical regional demographics after privacy protection is applied. The official poverty rate for the United States was 11.5% in 2022, and in the state of Kentucky, it was 16.5%, which places it 46th in a ranking of poverty rates in the United States [41]. For comparison, New Hampshire was ranked first and has the lowest poverty rate of 7.2% [41]. These example rates for the United States indicate areas experiencing differences of 5% to 7% in poverty rate, such as those reported in Table 2, represent vastly different socioeconomic dynamics. The extreme of this is illustrated in Table 2, where the largest difference between rates before and after obfuscation was 91.9% when records having relatively low poverty rates were assigned into areas having extreme poverty rates of 100% after obfuscation. The frequency of rate differences was substantially higher in the CCMEO data, which represents only Cook County, Illinois, and is home to Chicago, the third-largest urban area in the United States. In the 1990s, there were notable declines in the concentration of poverty in Chicago [42]. This decline, mixed with concerns regarding how gentrification has impacted the socioeconomic dynamics of Chicago, may explain why changes in poverty rate would occur at a higher frequency than in our larger OMOP data [43]. The impact of these shifts is important to understand when working with sensitive, protected health information and social determinants of health to correctly identify associations between place and health. As an example of poverty and health, women in high-poverty places are at greatest risk of being diagnosed with late-stage breast cancer [44]. Furthermore, different research studies may require different definitions of “neighborhood” when accessing socioeconomic statuses; for example, a person with multiple economic disadvantages may have a much narrower spatial range and limited social mobility.

We included small distances in our analysis to show that shifts occur even at very small distances. In obfuscation practice, small distances, such as 1 meter, would likely not be used due to the shifted point being too close to the original point and therefore not providing privacy; the balance between protecting privacy and protecting utility is context sensitive [21].

### Limitations

Different analytical applications may be variably sensitive to shifts in demographics; our method eliminates analytical concerns by avoiding shifts altogether. The caveat to our method is that constraining inherently limits the maximum distance a point can travel, which may not be suitable for all applications in terms of privacy requirements. For example, in Figure 2, pinwheel obfuscation moved points on average 482 meters away, while our geographically constrained pinwheel algorithm moved points on average 217 meters away. Some applications may not be suitable for point obfuscation; for example, research studies requiring distance to be preserved between subjects and waypoints such as hospitals or clinics may not tolerate any shifting of geographic coordinates.

### Public Health Impact

A previous evaluation of the pinwheel obfuscation method indicated that it had no major impact on geospatial analyses such as heat maps and hot spots [45]. However, we demonstrated that erroneous hot spots may be generated when analyzing deaths by suicide in Cook County, Illinois, including hot spots in uninhabitable areas. The issue presented in this paper is not a deficiency of the pinwheel technique, but a deficiency of data linkage using obfuscated points generated from any technique; if the variable that data linkage depends upon changes during obfuscation, then utility is harmed. We contend that any point obfuscation technique may be constrained to specific geographies.

An alternative solution could be that socioeconomic demographics are calculated using the real address data before data release, but data owners, especially state and local health agencies, have varying degrees of technical sophistication and may not be able to compute demographics. Our research group geocodes electronic health records on behalf of our local health care enterprise on campus, and we make the data available to any university researcher using our local data warehouse. Mobile applications, networking, and research with Internet of Things technology have explored privacy at different levels; our work is a step closer to context-aware point obfuscation within the epidemiology domain.

### Conclusions

A growing number of publicly available data sets are including precision geographic data for analysis. Our own work has explored decedent data published from open data portals for use in precision public health [14,33]. Point obfuscation can naturally shift a point into a different census-designated region; the regional differences before and after shifting highlight significantly different socioeconomic demographics. This is a natural consequence of moving a point and is not a weakness of the techniques themselves. We chose poverty as an example demographic due to its popularity in public health research; we also wish to explore the results of linking other census-level demographics. As future work, we will evaluate other techniques of point obfuscation and explore how these techniques may differ from those presented here. We show that it is possible to enhance point obfuscation by constraining where the new point may be placed; this ensures that the original point is obfuscated in a way that will not impact analyses depending upon the linkage to external region-based data.

## Abbreviations

ACS: American Community Survey
CCMEO: Cook County Medical Examiner’s Office
GIS: geographic information system
HIPAA: Health Insurance Portability and Accountability Act
ODMAP: Overdose Detection Mapping Application Program
OMOP: Observational Medical Outcomes Partnership
